# *Pectobacterium atrosepticum* Phage vB_PatP_CB5: A Member of the Proposed Genus ‘*Phimunavirus*’

**DOI:** 10.3390/v10080394

**Published:** 2018-07-26

**Authors:** Colin Buttimer, Alan Lucid, Horst Neve, Charles M. A. P. Franz, Jim O’Mahony, Dann Turner, Rob Lavigne, Aidan Coffey

**Affiliations:** 1 Department of Biological Sciences, Cork Institute of Technology, T12 P928 Cork, Ireland; colin.buttimer@mycit.ie (C.B.); alanlucid@gmail.com (A.L.); Jim.OMahony@cit.ie (J.O.); 2 Department of Microbiology and Biotechnology, Max Rubner-Institut, 24103 Kiel, Germany; horst.neve@mri.bund.de (H.N.); charles.franz@mri.bund.de (C.M.A.P.F.); 3 Department of Applied Sciences, University of the West of England, Bristol, Frenchay Campus, Coldharbour Lane, Bristol BS16 1QY, UK; dann2.turner@uwe.ac.uk; 4 Laboratory of Gene Technology, KU Leuven, 3001 Leuven, Belgium; rob.lavigne@kuleuven.be; 5 APC Microbiome Institute, University College, T12 YT20 Cork, Ireland

**Keywords:** *Pectobacterium atrosepticum*, *Autographivirinae*, ‘*Phimunavirus*’, bacteriophage, phage, *Podoviridae*

## Abstract

*Pectobacterium atrosepticum* is a phytopathogen of economic importance as it is the causative agent of potato blackleg and soft rot. Here we describe the *Pectobacterium* phage vB_PatP_CB5 (abbreviated as CB5), which specifically infects the bacterium. The bacteriophage is characterized in detail and TEM micrographs indicate that it belongs to the *Podoviridae* family*.* CB5 shares significant pairwise nucleotide identity (≥80%) with *P. atrosepticum* phages φM1, Peat1, and PP90 and also shares common genome organization. Phylograms constructed using conserved proteins and whole-genome comparison-based amino acid sequences show that these phages form a distinct clade within the *Autographivirinae*. They also possess conserved RNA polymerase recognition and specificity loop sequences. Their lysis cassette resembles that of *KP34virus*, containing in sequential order a U-spanin, a holin, and a signal–arrest–release (SAR) endolysin. However, they share low pairwise nucleotide identity with the type phage of the *KP34virus* genus, *Klebsiella* phage KP34. In addition, phage KP34 does not possess several conserved proteins associated with these *P. atrosepticum* phages. As such, we propose the allocation of phages CB5, Peat1, φM1, and PP90 to a separate new genus designated *Phimunavirus*.

## 1. Introduction

In the post-genomic era, the number of bacteriophage (phage) genomes being deposited into public databases such as the NCBI GenBank has substantially increased, due in part to the ever-decreasing cost of DNA sequencing. This growing quantity of genomic data has led to increasing insights into the evolutionary relationships between phages. Originally, taxonomic phage classification was based on morphology, nucleic acid composition, and physico-chemical characteristics [1]. More recently, classification has developed to the point where nucleotide and protein homology can be usefully employed to tease out phylogenetic relationships. This has led to the creation of subfamilies within *Myoviridae*, *Siphoviridae*, and *Podoviridae*, such as the subfamily *Autographivirinae*. This subfamily encompasses what was previously known as the T7 supergroup [2]. Key defining features of the subfamily *Autographivirinae* include the presence of a single RNA polymerase (RNAP) gene and a typical genomic organization with genes positioned on the Watson strand [3]. To date, this subfamily encompasses seven genera: the *T7virus*, *SP6virus*, *Phikmvvirus*, *Fri1virus*, *KP32virus*, *Pradovirus*, and *KP34virus* [2,4].

The bacterial genera *Pectobacterium* and *Dickeya*, often referred to collectively as the soft rot *Enterobacteriaceae*, are phytopathogens that cause economically important losses in a wide range of arable crops, thus potentially impacting food biosecurity. They are Gram-negative, facultative anaerobic rod-shaped cells that are typified by the production of extracellular pectinolytic enzymes during the infection of plants [5,6]. Within the last two years, a limited number of *Pectobacterium* and *Dickeya* phages have been reported whose genome sequences have been described to resemble phages of *KP34virus* and *Phikmvvirus*. The first of these to have their genome described was *Pectobacterium atrosepticum* phage Peat1 (accession no. KR604693) by Kalischuk et al. [7]. Phage φM1 (accession no. JX290549) was subsequently described by Blower et al. [8], after isolation and characterization by Toth et al. [9]. Related phages have also been described for *Pectobacterium carotovorum* subsp. *carotovorum* (namely phage PPWS1, accession no. LC063634.2) and *Dickeya* (phage BF25/12, accession no. KT240186.1) [10,11]. In addition, a *P. atrosepticum* phage, PP90 (accession no. KX278419.1), has been deposited to the public databases, as well as *P. carotovorum* subsp*. carotovorum* phage PP16 (accession no. KX278418). The latter two display high level of amino acid sequence similarity and possess a similar genomic organization of genes to phages of *KP34virus*.

In this study, we describe the newly isolated *P. atrosepticum* phage vB_PatP_CB5 (abbreviated as CB5). Phylogenetic analysis of its genome shows a close evolutionary relationship with *P. atrosepticum* phages φM1, Peat1, and PP90 (termed the PhiM1-like phages from here onwards in this article). Based on these findings, we propose the formation of the bacteriophage genus ‘*Phimunavirus*’ to formally classify these phages, with the *Pectobacterium* phage φM1 designated as the type phage.

## 2. Materials and Methods

### 2.1. Bacterial and Phage Propagation Conditions

To cultivate bacterial strains and phage, Lysogeny broth (LB), LB agar (1.5% w/v agar), and LB overlays (0.4% w/v agar) were employed. All cultures were grown at 25 °C. Phage CB5 was propagated using *P. atrosepticum* strain DSM 30186 using the methods described previously [12].

### 2.2. Phage Isolation

Phage CB5 was isolated using an enrichment method, as previously described [13]. Briefly, five grams of soil were weighed out and placed into 30 mL of LB broth along with 300 µL of overnight culture of *P. atrosepticum*. This sample was incubated for 18 h at 25 °C followed by centrifugation to remove particulate matter, after which the supernatant was filter-sterilized (0.45-µm pore-size filter, Sarstedt, Nümbrecht, Germany). The supernatant was spotted (10 µL) on top of the overlay, which had been seeded with an overnight culture of *P. atrosepticum*. The phage was isolated by picking off an individual plaque and then re-plating and re-isolating to generate a pure culture of the phage [14].

### 2.3. Host Range and General Characterization

The host range of the phage was tested by spotting serial dilutions (neat to dilution 10^−9^) of a phage suspension onto LB overlays seeded with the appropriate bacterial host, as described previously [15]. Bacteria strains used in host study are listed in Supplementary Information 1, Table S1. A number of the *Pectobacterium* strains utilized in this study have previously been described by Buttimer et al. [13].

A similar approach to the one step growth curve assay described previously was used [16,17]. The host bacteria (strain DSM 30186) were grown to an OD_600_ of 0.20–0.23 (ca. 1 × 10^8^ colony forming units (CFU)/mL), followed by centrifugation of 2 mL in a microfuge to pellet bacteria. The pellet was resuspended in 1 mL of phage suspension to yield an approximate multiplicity of infection (MOI) of 5 × 10^−4^ following incubation at 25 °C for 1 min. This was then centrifuged to pellet bacteria, and the supernatant was removed, thus separating bound from unbound phages. The bacterial pellet with bound phage was then resuspended in 10 mL of LB and incubated aerobically in a water bath at 25 °C with agitation at 60 rpm. At 5-min intervals, aliquots were removed to measure phage titer by the overlay method. Based on the number of PFU/mL of each replicate, the latent period and the burst size were determined by dividing the average PFU/mL of the latent period by the average PFU/mL of the last four time points of the experiment.

### 2.4. Transmission Electron Microscopy

Prior to electron microscopic analysis, phages were purified by CsCl density gradient centrifugation as previously described [13]. Phages adsorbed to freshly prepared ultra-thin carbon film were: (1) treated with 1% (v/v) EM-grade glutaraldehyde (20 min) for fixation; (2) negatively stained with 1% (w/v) uranyl acetate; and (3) subsequently analyzed using a Tecnai 10 transmission electron microscope (FEI Thermo Fisher, Eindhoven, The Netherlands) at an acceleration voltage of 80 kV. Digital micrographs were acquired with a MegaView G2 CCD camera (EMSIS, Muenster, Germany).

### 2.5. DNA Isolation and Sequencing

DNA extraction was performed as previously described [18]. Briefly, free nucleic acids were removed from phage lysates (ca. 1 × 10^10^ PFU/mL) with DNase and RNase, treated with 10% SDS and proteinase K followed by DNA extraction with phenol:chloroform:isoamyl alcohol (25:24:1 v/v) and chloroform:isoamyl alcohol (24:1 v/v). Prior to sequencing, DNA quality and quantity were assessed by using both a Nanodrop (ND-1000, Thermo Fisher, Waltham, MA, USA) and by visualization after agarose gel electrophoresis. DNA sequencing was outsourced to GATC Biotech (Konstanz, Germany). To conduct sequencing, DNA libraries were first created by DNA fragmentation, adapter ligation followed by a size selection and amplification. DNA libraries were then measured and quantified on a fragment analyzer before sequencing with 2 × 300 bp paired-end reads using the Illumina Hiseq system (Illumina, San Diego, CA, USA). The *de novo* assembly was performed using default parameters with CLC Genomics Workbench v8.0 (Qiagen, Aarhus, Denmark).

### 2.6. Bioinformatic Analysis

Open reading frames (ORFs) of CB5 were predicted with GLIMMER [19] and GenmarkS [20]. Functional inferences for predicted ORF gene products were obtained by searches conducted using BLASTP (http://blast.ncbi.nlm.nih.gov/Blast.cgi?PAGE=Proteins), Pfam (http://pfam.xfam.org/search#tabview=tab1; [21]), InterProScan (https://www.ncbi.nlm.nih.gov/pmc/articles/PMC3998142/; [22]) and HHpred (https://toolkit.tuebingen.mpg.de/#/tools/hhpred; [23]). Transmembrane domains and lipoprotein cleavage signal were identified using TMHMM v.2 (http://www.cbs.dtu.dk/services/TMHMM/; [24]) and LipoP v.1 (http://www.cbs.dtu.dk/services/LipoP/; [25]), respectively. The molecular weight of the predicted ORFs was estimated using the batch protein molecular weight determination of the sequence manipulation suite (http://www.bioinformatics.org/sms2/protein_mw.html). The presence of transfer RNA genes was investigated with the use of tRNAscan-SE (http://lowelab.ucsc.edu/tRNAscan-SE/; [26]) and ARAGORN (http://130.235.46.10/ARAGORN/; [27]).

### 2.7. Comparative Genomics

To determine shared proteins among phage proteomes, CoreGenes 3.5 (http://binf.gmu.edu:8080/CoreGenes3.5/; [28]) was used. Translated ORFs from phage φM1 were searched against hidden Markov model profiles downloaded from the prokaryotic Virus Orthologous Groups (pVOGs) database (PMID: 27789703, [29]) using hmmscan (PMID: 22039361, [30]) with an E-value cutoff of 1 × 10^−3^. Matches to pVOG profiles were considered significant at an E-value of ≤1 × 10^−15^ and ≥35% coverage of the profile HMM. The linear genomic comparison maps were created with the use of either BLASTN or TBLASTX, to determine similarity, and then visualized with Easyfig [31]. Phylograms were generated based on the amino acid sequences of the major capsid protein of phage CB5 and 52 members of *Autographivirinae* (Supplementary Information 1, Table S2) using MEGA7 [32], applying MUSCLE for sequence alignment [33] with the construction of phylograms using the maximum likelihood (ML) method based on the Whelan and Goldman substitution model [34], with the robustness of the trees assessed by bootstrapping (1000). VICTOR was employed using all pairwise comparisons of the amino acid sequences (same phages as described previously) which employs the Genome-BLAST Distance Phylogeny (GBDP) method [35] under settings recommended for prokaryotic viruses [36]. The resulting intergenomic distances (including 100 replicates each) were used to infer a balanced minimum evolution tree with branch support via FASTME, including SPR postprocessing [37] for each of the formulas D0, D4, and D6, respectively. The trees were rooted at the midpoint [38] and visualized with FigTree [39]. Taxon boundaries at the species, genus, subfamily, and family level were estimated with the OPTSIL program [40], recommended clustering thresholds [36], and an F value (fraction of links required for cluster fusion) of 0.5 [41]. The heat map comparing the genomes of phage CB5 and 31 phages of *Autographivirinae* was generated using Gegenees utilizing TBLASTX, with accurate parameters (fragment length: 200 bp; step size: 100 bp, threshold set to 5%) [42]. Alignment of the RNAP proteins for the examination of catalytic active residues and residues of the recognition and specificity loop was conducted using MUSCLE on MEGA7.

### 2.8. Accession Number

The genome sequence of phage CB5 was submitted to GenBank under accession number KY953156.

## 3. Results

### 3.1. Isolation, Host Range, Growth Characteristics and Morphology

**Figure 1:**
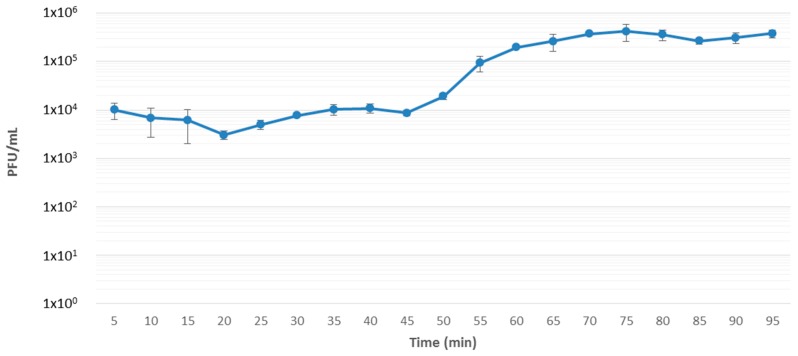
One-step growth curve of *Pectobacterium* phage vB_PatP_CB5 infection of host bacterium DSM 30186. Assay was independently repeated in triplicate and the results were averaged.

Phage CB5 was isolated from soil samples collected from potato grading machinery on a farm in Co. Cork, Ireland, during the year 2013, as mentioned previously [13]. Host range was determined on 31 bacterial strains from five different species belonging to soft rot *Enterobacteriaceae*, namely *P. atrosepticum* (19 strains), *P. carotovorum* subsp. *carotovorum* (four strains), *Dickeya chrysanthemi* bv. *chrysanthemi* (one strain), *Dickeya dianthicola* (three strains), and *Dickeya solani* (four strains). The phage possesses a narrow host range, in that it is only capable of forming plaques on the phage’s host strain (DSM 30186) and two other strains of *P. atrosepticum* (Table 1)*.* Additionally, spot tests showed that the phage had an inhibitory effect on 15 of the other 16 strains of *P. atrosepticum* tested with the observation of zones of clearing at high phage titers despite the absence of distinct plaques. No plaque formation or inhibition was detected for any other bacterial species tested. A similar narrow host range has also been reported for PhiM1-like *Pectobacterium* phage φM1, with a infectivity range confined to a small number of *P. atrosepticum* strains [9]. This limited host range has also been observed among phages of *KP34virus* and *Phikmvvirus* [43,44]. On the propagating host strain DSM 30186, CB5 was found to produce clear plaques with an approximate diameter of 3 mm (Supplementary Information 1, Figure S1). One-step-growth curve assay, under standard conditions using LB medium, demonstrated that phage CB5 possessed a latent period of 45 min with an approximate burst size of 44 ± 8 PFU/cell (Figure 1).

**Figure 2:**
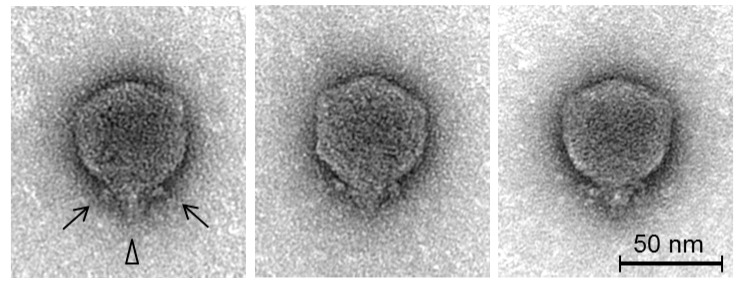
Transmission electron microscopy of negatively stained *Pectobacterium* phage CB5 using 1% (w/v) uranyl acetate. Triangle indicates the short conical tail structure, and arrows indicate short appendages (whiskers) beneath the capsid. Scale bars represent 50 nm.

Examination of the morphology of the phage by transmission electron microscopy (Figure 2) shows it can be classified as a member of the *Podoviridae* family. It features a C1 morphotype [45] with an icosahedral head (63.1 ± 3.6 nm in diameter, *n* = 25) with clearly distinguishable hexagonal outlines and a short non-contractile tail (13.1 ± 1.8 nm, *n* = 11), and short appendices (length: ca. 10.1 ± 1.7 nm, *n* = 10) visible at the head/tail connection site. These head and tail dimensions are consistent with previously reported phages of *KP34virus* and *Phikmvvirus* [44,46]. The phage was formally named in accordance with the nomenclature set out by Kropinski et al. [47].

### 3.2. General Genome Information of CB5

The genome sequence size obtained for phage CB5 is 44,262 bp (coverage >1000×) and examination of sequence reads indicated the presence of direct terminal repeats (DTRs) with an estimated size of 287 bp. This estimation is based on the identification of a localized region with more than double the read depth compared to average read depth across the whole genome (Supplementary Information 1, Figure S2). This approach to detect DTRs has also been applied to a number of other phages [48,49,50]. Additionally, the average G + C% content of its genome was found to be 49%. This is close to the range that is typically expected for its host bacterium, *P. atrosepticum*, at 50–51% [51,52].

The genome of CB5 was predicted to contain 60 ORFs. These, apart from one, were found to read in the 5’ to 3’ direction, with GC skew correlating well with transcription [53]. Based on analysis using a combination of BLASTP, InterProScan, and HHpred, putative functions to 33 of the 60 predicted ORFs (55%) were assigned. These gene products can be categorized into DNA replication, virion structure, and host lysis functions (Supplementary Information 2, Table S3). Of the assigned ORFs, five are predicted to encode homing endonucleases of the HNH family (CB5_17, 23, 29, 41, 48) (IPR003615). No integrase, excisionase, nor repressor genes were detected, suggesting the phage has an exclusively lytic lifecycle. Furthermore, no tRNA genes were identified.

### 3.3. Comparative Genomics of PhiM1-Like Phages

The four phages within the proposed genus ‘*Phimunavirus*’ possess genomes of similar size that share a high degree of sequence similarity and share a large number of conserved proteins (Table 2). Genome sizes (excluding DTRs) range from 43,534 bp (φM1) to 45,633 bp (Peat1), with nucleotide pairwise identity between the four phages ranging from 82% to 86% (BLASTN). Total ORF numbers range from 52 (φM1) to 61 (Peat1), with CoreGenes analysis showing that they collectively share a minimum of 39 proteins, including 32 which were affiliated to a known pVOG (Table 3). These coding sequences are spread across the entire genome and are not associated/limited to particular genomic modules. Of the four phages, only φM1 possesses a tRNA gene (for isoleucine). G + C content among the four phages is highly similar, ranging from 48.7% to 49.2%. Additionally, these phages show limited similarity to *Klebsiella* phage KP34 (7% to 9% identity). Indeed, CoreGenes shows that phage KP34 shares 29 proteins with the PhiM1-like phages (Table 3). The major variations of conserved proteins of phage KP34 with these phages are five hypothetical proteins located in the DNA replication and nucleotide metabolism region. Also, additional hypothetical proteins were identified immediately downstream of the genes encoding the RNAP and large terminase proteins, as well as the ORFs for their predicted holin and tail spike.

**Figure 3:**
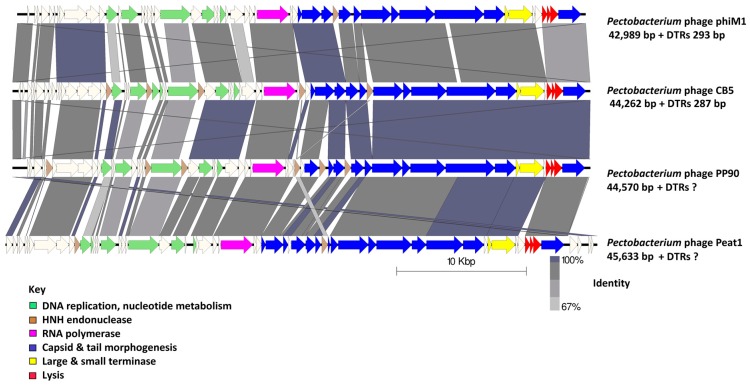
Comparison of the genomes of the phages that form the proposed genus of ‘*Phimunavirus*’. *Pectobacterium* phage CB5 and *Pectobacterium* phages φM1, Peat1, and PP90 are shown using currently available annotations from Genbank, employing BLASTN and visualization with Easyfig. The genome maps display arrows indicating locations and orientation of ORFs among different phage genomes. Arrows have been color-coded describing their predicted roles (see key), and shading between the genome maps indicates the level of identity. Phage DTRs of unknown length marked with “?”.

**Figure 4:**
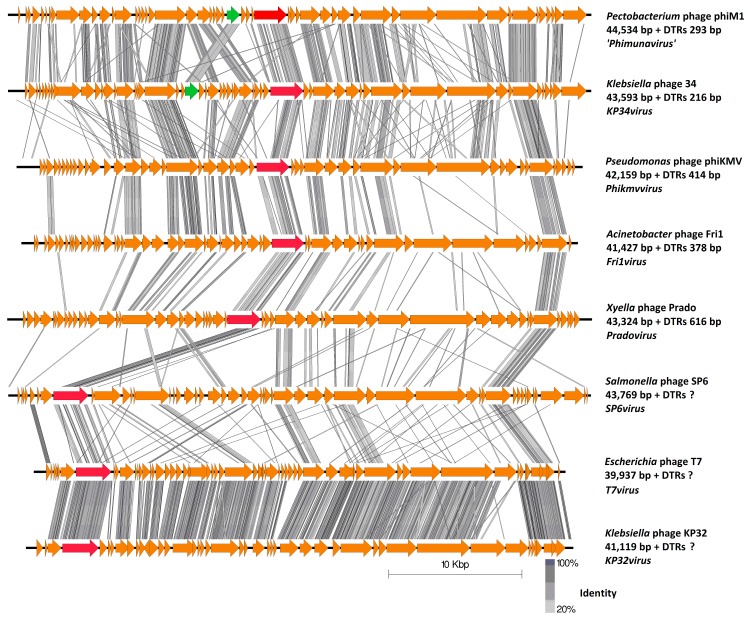
Pairwise comparison of the type phages representing six genera of the subfamily *Autographivirinae*; *KP34virus*, *Phikmvvirus*, *Frivirus*, *SP6virus*, *Pradovirus* and *T7virus*, and against *Pectobacterium* phage φM1. Genomic maps were created using currently available annotation from Genbank with comparisons employing TBLASTX and visualization with Easyfig. The genome maps display arrows indicating locations and orientation of ORFs. Lines between genome maps indicate the level of identity. The ORF of a conserved protein shared between φM1 and KP34 that does not mirror genomic position is color-coded green. The ORF encoding the RNAP shared between genera of *Autographivirinae* has been color-coded red. Phage DTRs of unknown length marked with “?”.

The gene order is highly conserved among the PhiM1-like phages (Figure 3). Their genome architecture is arranged so that the predicted early and middle gene regions end with a RNAP gene (*CB5_39*), with ORFs within these regions involved in DNA replication and nucleotide metabolism, but also expected to be involved in host takeover. The position of ORFs for RNAP in the PhiM1-like phages is shared with phages of the genera *KP34virus*, *Fri1virus*, *phiKMVvirus and Pradovirus*, but not with those of the genera *T7virus*, *SP6virus*, and *KP32virus* where the RNAP is situated at the early gene region (Figure 4). The late gene region of the PhiM1-like phages is associated with virion morphogenesis and host lysis roles. Gene order between PhiM1-like phages and KP34-like phages is highly conserved apart from the position of an ORF encoding a conserved protein (CB5_36, PhiM1_27, AXI77_gp27, PP90_28) possessing a calcineurin-like phosphoesterase domain (IPR004843) (Figure 4).

### 3.4. Phimunavirus Evolutionary Position within the Autographivirinae

**Figure 5:**
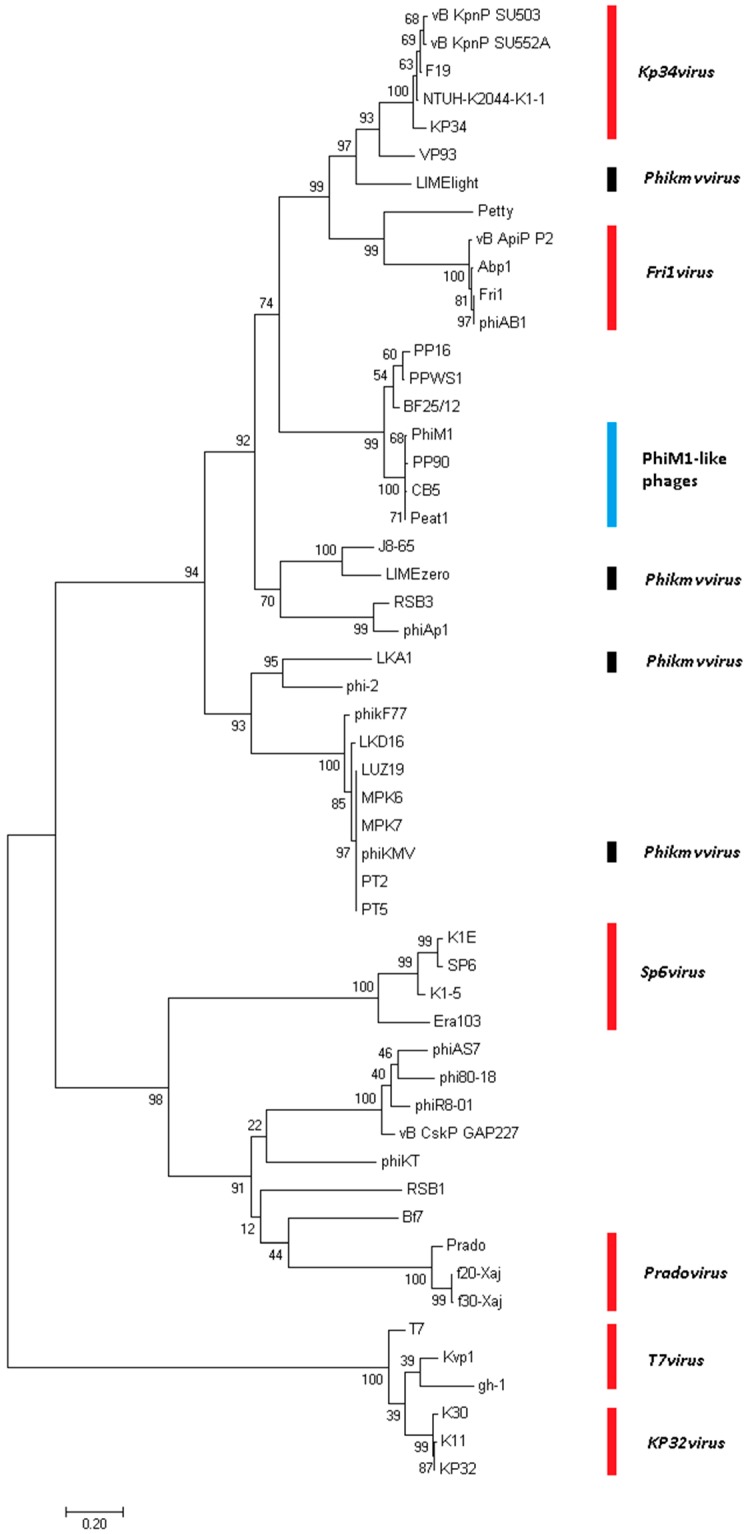
Phylogenetic analyses of amino acid sequences of the major capsid proteins (log likelihood = 13,809.83) of *Pectobacterium* phage CB5 and 52 members of the *Autographivirinae* subfamily using maximum likelihood (Whelan and Goldman substitution model), with 1000 bootstrap replicates. Members of the *T7virus*, *SP6virus*, *KP34virus*, *Frivirus*, *Pradovirus*, *KP32virus* are illustrated.

**Figure 6:**
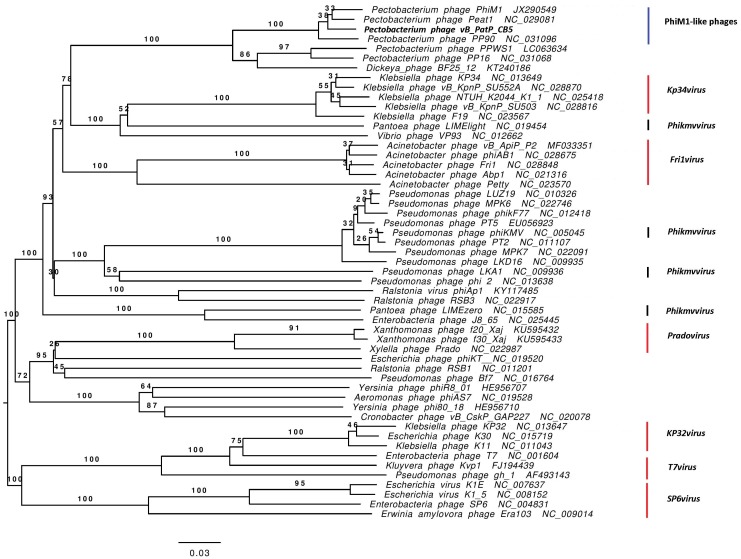
VICTOR-generated phylogenomic Genome-BLAST Distance Phylogeny (GBDP) trees of *Pectobacterium* phage CB5 and 52 members of the *Autographivirinae* subfamily inferred using the formula D4 and yielding average support of 71%. The numbers above branches are GBDP pseudo-bootstrap support values from 100 replications. Members of the *T7virus*, *SP6virus*, *KP34virus*, *Frivirus*, *Pradovirus*, and *KP32virus* are illustrated.

**Figure 7:**
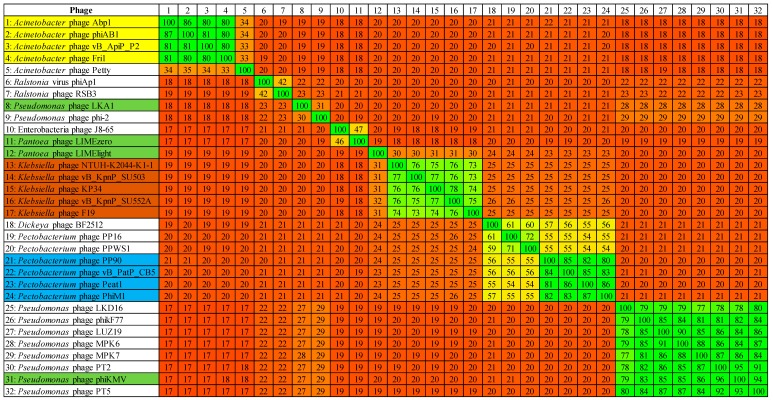
TBLASTX heat map generated using Gegenees with accurate parameters—fragment length: 200 bp; and step size: 100 bp with the threshold set to 5%. The map includes the genomes of 32 phages of *Autographivirinae* with phages representing the genera *Fri1virus* (yellow), *KP34virus* (brown), *Phikmvvirus* (green) and the proposed genus of ‘*Phimunavirus*’ (blue). Plot colors reflect the identity, ranging from low (red) to high (green).

To determine the PhiM1-like phages’ evolutionary relationship to other phages within the *Autographivirinae*, a phylogenetic analysis was conducted using the amino acid sequence of the major capsid protein (Figure 5). The resulting phylogram showed that the PhiM1-like phages form their own clade on a branch containing an additional clade representing the *P. carotovorum* subsp. *carotovorum* phages PP16 and PPSW1 and *Dickeya* phage BF25/12. The branch that these phages form was found to be positioned close to phages of the *KP34virus* genus along with sister groups consisting of *Vibrio* phage VP93 and the *Pantoea* phage LIMElight, which have previously been described to possess a close evolutionary relationship to the *KP34virus* members [44]. This analysis was performed with the head–tail connector protein, a large terminase subunit, and tail tubular proteins A and B. These analyses produced tree exhibiting similar relationships, albeit with weaker bootstrap support values (Supplementary Information 1, Figure S3). Whole-genome comparison based on amino acid sequences was performed using VICTOR and the resulting phylogram (formula D4, yielding average support of 71%) presented a similar conclusion as that based on major head protein sequence (Figure 6). Additionally, analysis using VICTOR could cluster these 53 phage genomes into 13 genera and four subfamilies, with PhiM1-like phages being placed in their own genera with *P. carotovorum* subsp. *carotovorum* phages PP16 and PPSW1 and *Dickeya* phage BF25/12 (Supplementary Information 1, Table S4). Further analysis using Gegenees (TBLASTX), based on protein similarity, indicate the PhiM1-like phages form a clade with high identity values of ≥80%. Additionally, as seen in the phylograms, it was observed that *P. carotovorum* subsp. *carotovorum* phages PP16 and PPSW1 and *Dickeya* phage BF25/12 share an evolutionary relationship with PhiM1-like phages (identity values ≥54%) (Figure 7).

### 3.5. RNAP of the PhiM1-Like Phages

The RNAP protein has been used as a feature to establish genera within the subfamily of *Autographivirinae*. Within the amino acid sequence of the RNAP of phage T7, the key catalytic residues Asp537, Lys631, Tyr639 and Asp812 and the regions of functional importance, namely the recognition loop (93–101 aa) and the specificity loop (739–770 aa) are generally well conserved among different clades within *Autographivirinae* [43,44]*.* Analysis of PhiM1-like phages shows that they all have the catalytic residues Asp537, Lys631 and Asp812. Comparisons of the recognition loop and specificity loop of these phages show that they are vastly different to φKMV, with the recognition loop of KP34 and Fri1 possessing a small resemblance to that of these phages (Table 4). Furthermore, sequence variation is evident between the PhiM1-like phages and the closely related *P. carotovorum* subsp. *carotovorum* phages PP16 and PPSW1 and *Dickeya* phage BF25/12.

### 3.6. Early Gene Region

Early region ORFs are those that are expected to be transcribed immediately upon internalization of phage DNA within the host. *In silico* analysis of ORFs for hypothetical proteins within this region is typically limited (in the context of determining their functions) for the majority of phages due to their vast diversity. However, it can be expected that these ORFs are involved in the redirection of host proteins to a role in the phage infection cycle through stimulation or inhibition of protein–protein interactions [54]. Here, we define the early genes among the PhiM1-like phages as those positioned before the DNA primase ORF (Figure 3). Six ORFs were found to be conserved within this region using CoreGenes (Supplementary Information 1, Table S5). Of these six ORFs, only one could be assigned the putative function of a peptidase (IPR007484).

### 3.7. DNA Replication, Repair, and Related Metabolism

PhiM1-like phages encode ORFs for proteins involved in the replication and repair of DNA (Supplementary Information 1, Table S6), including a primase, helicase, DNA polymerase, endonuclease VII and a putative 5′ exonuclease. The order of these ORFs is conserved among the genomes of these phages with variation existing in the context of the presence of ORFs for hypothetical proteins and homing endonucleases among the primase, helicase, DNA polymerase and endonuclease VII. Additional variations identified were that CB5 possesses an ORF encoding a putative nucleatidyl transferase, which is absent among other PhiM1-like phages, and that CB5 and Peat1 lack an ORF encoding a putative polynucleotide 5′-kinase/3′-phosphatase that is shared between PP90 and φM1.

### 3.8. Structure-Related Genes

Discussion of these genes also includes those specifying large and small terminase (maturase) proteins which play a role in the packaging of DNA into the phage capsid. A total of 12 ORFs predicted to be involved in virion morphogenesis are shared among the PhiM1-like phages (Supplementary Information 1, Table S7). These include a head–tail connector protein, a virion scaffolding protein, major capsid protein, tail tubular proteins A and B, internal virion proteins A, B and C, a tail fiber, and a tail spike. The order in which the ORFs for these proteins occur in the genomes of these phages is highly conserved. Minor differences within this synthetic region were due to the presence of homing endonucleases among the ORFs or in some cases splitting ORFs, for example the head–tail connector protein of PP90 (PP90_39, 40). Additionally, it was noted that some structural proteins were encoded by split ORFs without the presence of homing endonucleases, as seen for the major capsid protein (AX177_gp38, 39), tubular protein A (AX177_gp41, 42) and internal virion protein C of Peat1 (AX177_gp46, 47).

The predicted tail spike protein of these phages possesses the P22 tailspike domain (IPR015331). The P22 tailspike is characterized by the right-handed beta helix architecture first observed for pectate lysase [55]. This protein is capable of breaking down saccharides upon binding to host cell surface [56], which may be the case also for the PhiM1-like tail spike. The internal virion protein B of these phages may also possess enzymatic activity, with HHpred analysis indicating homology to phage proteins with lysozyme activity (best hit against PhiM1_42; *Escherichia* phage P1 endolysin Lyz, PDB accession no. 1XJU_A). This suggests that this protein may play a role in the breakdown of cell wall peptidoglycan during injection of phage genomic DNA into its host cell, like Gp16 of phage T7 [57].

### 3.9. Lysis Cassette of PhiM1-Like Phages Resembles That of KP34virus

**Figure 8:**
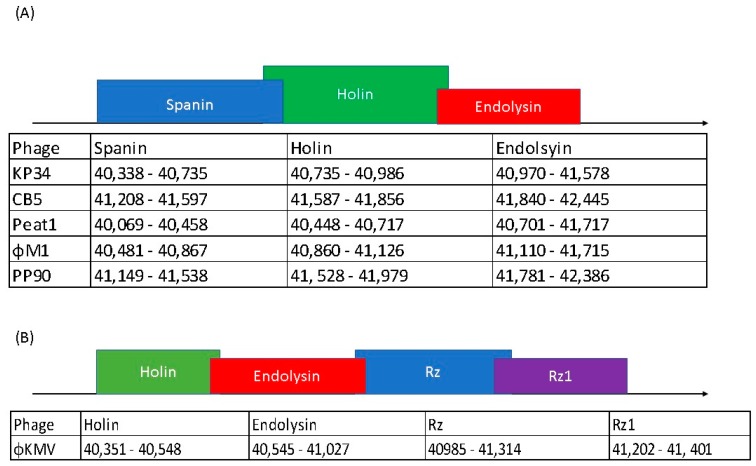
Lysis cassette scheme of (**A**) *Klebsiella* phage KP34 and PhiM1-like phages compared to (**B**) *Pseudomonas* phage φKMV.

The PhiM1-like phages possess three proteins predicted to cause host lysis. These are arranged in a conserved order: a spanin, a holin and an endolysin (Supplementary Information 1, Table S8). The endolysins of these phages are predicted to possess a N-terminal transmembrane domain with lysozyme domain (IPR023347), indicating a likely function as a signal–arrest–release (SAR) endolysin, similar to that described for *Pseudomonas* phage φKMV [58]. SAR endolysins use the host *sec* translocon system to enable their transport to the cell’s inner membrane. The most likely holin of these phages is the pin-hole holin variety. These can provide narrow channels for ion movement causing membrane depolarization and activation of the SAR endolysin resulting in the degradation of cell wall peptidoglycan [58,59]. Spanins are proteins responsible for the destruction of the outer membrane of Gram-negative hosts allowing phage progeny release. The predicted spanin of these phages is comprised of a single protein with an N-terminal outer-membrane lipoprotein signal and a C-terminal transmembrane domain, classifying them of the u-spanin variety [60]. The lysis cassette configuration of the PhiM1-like phages resembles that of *Klebsiella* phage KP34, sharing the same gene variations with the type phage φKMV of *Phikmvvirus*. The spanin of φKMV is composed of a two protein component system, an i-spanin integral cytoplasmic membrane protein (Rz), and an o-spanin outer membrane lipoprotein (Rz1) [60,61]. Variation also exists with the order of occurrence of the genes for these proteins (holin, SAR endolysin, Rz, and Rz1) in the lysis cassette of φKMV in comparison to the PhiM1-like phages and phage KP34 (Figure 8).

## 4. Discussion

*In silico* analysis shows that the *Pectobacterium* phage CB5 belongs to a distinct group of phages (φM1, Peat1 and PP90) that infect *P. atrosepticum* and that can be classified as members of the subfamily *Autographivirinae*. In this article, we propose the creation of the genus ‘*Phimunavirus*’ to encompass these phages, the genus name is derived from the allocated type phage φM1, the first representative of these phages to be described. This proposal is supported due to these phages sharing a high DNA pairwise identity of ≥80% (BLASTN), with a highly conserved gene order (Figure 3) and a shared protein content of ≥60% (CoreGenes). They also form a distinct clade when compared to other phages of *Autographivirinae* on phylograms based on their major capsid protein sequences (Figure 5) and whole-genome comparison based on amino acid sequences employing VICTOR (Figure 6). Furthermore, Gegenees analysis (TBLASTX) based on amino acid sequences also indicates the existence of this clade (Figure 7). Additionally, the recognition and specificity loop of the RNAP between these phages is highly conserved (Table 4). These phages share a number of characteristics with members of the *KP34virus* genus. Such as a similar arrangement of genes (Figure 4) like that of the lysis cassette, arranged sequentially in the order of a u-spanin, holin and SAR endolysin (Figure 8). However, DNA pairwise identity of these phages with the type phage, *Klebsiella* phage KP34, is low at 7–9%. In addition, CoreGenes analysis showed that the PhiM1-like phages also possess a number of conserved proteins not shared with KP34 (39 vs. 29 proteins, respectively) (Table 3). Furthermore, the amino acid sequence of the recognition and specificity loop of the RNAP of KP34 differs to that of PhiM1-like phages (Table 4). Interestingly, phylograms of the conserved proteins (Figure 5 and Supplementary Information 1, Figure S3) and whole-genome comparison based on the amino acid sequence (Figure 6) show a more closely placed evolutionary relationship of *Vibrio* phage V93 and *Pantoea* phage LIMElight with the *KP34virus* genus than with the PhiM1-like phages. Features that exclude these phages from being incorporated into *KP34virus* genus were that phage V93 possesses a distinct lysis cassette and encodes two tail proteins, while phage LIMElight encodes a tail fiber protein with greater similarity to phages of *SP6virus* than to that of KP34 [44]. 

It is clear that *P. carotovorum* subsp. *carotovorum* phages PP16 and PPSW1 and *Dickeya* phage BF25/12 share a close evolutionary relationship with PhiM1-like phages, sharing DNA pairwise identity of between 47% and 55%. They also form a clade that sits on the same branch as that of PhiM1-like phages on phylograms comparing the major capsid protein and whole-genome comparison based on amino acid sequences of phages of *Autographivirinae* (Figure 5 and Figure 6). Furthermore, Gegenees analysis (TBLASTX) shows a shared identity of between 54% and 60% (Figure 7). However, the relationship is more distant than that between phages of the suggested ‘*Phimunavirus*’ genus. Thus, casting doubt on whether they should be placed in the genus*,* we have chosen to exclude phages PP16, PPSW1, and BF25/12 from the genus at this point in time until the availability of more data on related phages.

Not all *Pectobacterium* phages reported to date that have been classified as belonging to *Autographivirinae* resemble *Pectobacterium* phage φM1. For example, *Pectobacterium carotovorum* subsp. *carotovorum* phage PP2 possesses homology to *Cronobacter* phage vB_CsaP_Gap277, a phage that has been identified to represent a distinct genus within *Autographivirinae* [62,63].

Finally, it has been commented that the split of the *Phikmvvirus* genus into genera better reflecting evolutionary relationships is to date incomplete [64]. This concern was highlighted in phylograms constructed in this study with the observation that the phage LIMElight, which is currently classified as a member of the *Phikmvvirus*, is in fact placed between the genera *Fri1virus* and *KP34virus* (Figure 5 and Figure 6)*.* Since the creation of the *Autographivirinae* subfamily, many more phages related to it have been sequenced. It is clear from the phylograms constructed in this study that a taxonomic reassessment of these phages is required to adequately reflect their genomic diversity.

## Figures and Tables

**Table 1:** Host range of *Pectobacterium* phage vB_PatP_CB5 (CB5) on 31 strains of the soft rot *Enterobacteriaceae*, determined by spot testing with serial dilutions of phage.

Species	Strain	Sensitivity
*Pectobacterium atrosepticum*	DSM 18077 (type strain)	++
	DSM 30184	+
	DSM 30185	+
	DSM 30186	++ *
	CB BL1-1	+
	CB BL2-1	+
	CB BL3-1	+
	CB BL4-1	+
	CB BL5-1	+
	CB BL7-1	+
	CB BL9-1	+
	CB BL11-1	+
	CB BL12-2	++
	CB BL13-1	+
	CB BL14-1	+
	CB BL15-1	−
	CB BL16-1	+
	CB BL18-1	+
	CB BL19-1	+
*Pectobacterium carotovorum* subsp. *carotovorum*	DSM 30168 (type strain)	−
	DSM 30169	−
	DSM 30170	−
	CB BL19-1-37	−
*Dickeya chrysanthemi* bv *chrysanthemi*	LMG 2804	−
*Dickeya dianthicola*	PD 482	−
	PD 2174	−
	GBBC 1538	−
*Dickeya solani*	sp. PRI 2222 (D36)	−
	LMG 25865 (D10)	−
	GBBC 1502	−
	GBBC 1586	−

Results recorded as ++, sensitive; +, presence of clear spot with no plaque formation; −, no infection; * host strain of phage.

**Table 2:** Properties of the seven phages belonging to the proposed genus of ‘*Phimunavirus*’. ORF: open reading frame; DTR: direct terminal repeat.

Phage	Genome Size (bp)	DTRs (bp)	G + C Content, %	ORFs	tRNA	DNA Identity, % *	Homologous Proteins, % **
φM1	43,534	293	49.18	52	1	100	100
CB5	44,262	287	48.98	60	0	84	73
Peat1	45,633	NA	48.86	61	0	86	87
PP90	44,570	NA	48.89	56	0	86	80

* DNA identity in comparison to φM1 using BLASTN; ** Number of homologous proteins in comparison to φM1 using CoreGenes; NA, not available.

**Table 3:** Thirty-nine conserved genes among phages (φM1, Peat1, CB5, PP90) of the proposed genus ‘*Phimunavirus*’, as determined by CoreGenes, and their details with regard to the type phage φM1. Conserved proteins of these phages shared with *Klebsiella* phage KP34 are highlighted in bold. Additionally, prokaryotic Virus Orthologous Groups (pVOGs) determined from translated ORFs of φM1 of these conserved proteins are presented.

No.	Product	φM1 Accession No.	Φm1 Locus Tag	pVOG
**1**	**hypothetical protein**	**AFQ22488.1**	**PhiM1_03**	**VOG6006**
**2**	**hypothetical protein**	**AFQ22489.1**	**PhiM1_04**	**VOG1073**
**3**	**hypothetical protein**	**AFQ22493.1**	**PhiM1_08**	**VOG5528**
**4**	**hypothetical protein**	**AFQ22494.1**	**PhiM1_09**	**-**
**5**	**putative peptidase**	**AFQ22495.1**	**PhiM1_10**	**VOG5332**
**6**	**hypothetical protein**	**AFQ22496.1**	**PhiM1_11**	**VOG5704**
**7**	**putative DNA primase**	**AFQ22497.1**	**PhiM1_12**	**VOG4551**
**8**	**putative DNA helicase**	**AFQ22499.1**	**PhiM1_14**	**VOG0025**
9	hypothetical protein	AFQ22501.1	PhiM1_16	-
10	hypothetical protein	AFQ22503.1	PhiM1_18	-
**11**	**DNA polymerase**	**AFQ22505.1**	**PhiM1_20**	**VOG0026**
**12**	**hypothetical protein**	**AFQ22506.1**	**PhiM1_21**	**VOG1076**
**13**	**DNA exonuclease**	**AFQ22507.1**	**PhiM1_22**	**VOG0028**
14	hypothetical protein	AFQ22508.1	PhiM1_23	-
**15**	**DNA endonuclease VII**	**AFQ22510.1**	**PhiM1_25**	**VOG8238**
**16**	**putative metallophosphoesterase**	**AFQ22512.1**	**PhiM1_27**	**VOG1606**
17	hypothetical protein	AFQ22514.1	PhiM1_29	VOG1254
18	hypothetical protein	AFQ22515.1	PhiM1_30	VOG9679
**19**	**putative RNA polymerase**	**AFQ22516.1**	**PhiM1_31**	**VOG0019**
20	hypothetical protein	AFQ22517.1	PhiM1_32	VOG1406
21	hypothetical protein	AFQ22518.1	PhiM1_33	VOG9202
**22**	**putative structural protein**	**AFQ22519.1**	**PhiM1_34**	**VOG8332**
**23**	**putative head–tail connector protein**	**AFQ22520.1**	**PhiM1_35**	**VOG0030**
**24**	**putative scaffolding protein**	**AFQ22521.1**	**PhiM1_36**	**VOG0031**
**25**	**putative endonuclease**	**AFQ22522.1**	**PhiM1_37**	**-**
**26**	**putative capsid protein**	**AFQ22523.1**	**PhiM1_38**	**VOG4572**
**27**	**putative tail tubular protein A**	**AFQ22524.1**	**PhiM1_39**	**VOG4592**
**28**	**putative tail tubular protein B**	**AFQ22525.1**	**PhiM1_40**	**VOG0034**
**29**	**putative internal core protein A**	**AFQ22526.1**	**PhiM1_41**	**VOG1080**
**30**	**putative internal core protein B**	**AFQ22527.1**	**PhiM1_42**	**VOG3794**
**31**	**putative internal core protein C**	**AFQ22528.1**	**PhiM1_43**	**VOG0038**
**32**	**putative tail fiber protein**	**AFQ22529.1**	**PhiM1_44**	**-**
**33**	**putative DNA maturase A**	**AFQ22530.1**	**PhiM1_45**	**VOG0041**
**34**	**putative DNA maturase B**	**AFQ22531.1**	**PhiM1_46**	**VOG4544**
35	hypothetical protein	AFQ22532.1	PhiM1_47	-
**36**	**putative Rz1A protein**	**AFQ22534.1**	**PhiM1_49**	**VOG1082**
37	putative holin	AFQ22535.1	PhiM1_50	VOG0765
**38**	**endolysin**	**AFQ22536.1**	**PhiM1_51**	**VOG4565**
39	phage tail spike protein	AFQ22537.1	PhiM1_52	VOG4640

**Table 4:** Alignment of the recognition and specificity loops of the RNAP of PhiM1-like phages to phages φKMV and KP34. Underlined amino acids/residues show sites of substitutions in comparison to φM1.

Phage	Recognition Loop	Specificity Loop
φKMV	HQEAKAAGPAAKL	EEVRVRLRAEAVEYVTLYEAK-DE
KP34	MRNVKAPGIGGKY	EEVRVRIDCMNLSAVLVHNRDFKT
Fri1	VKKQKIRGVGGKY	VTKTVAIRSMGINNIAYRYPD-NQ
φM1	ICSKGTRGVGGKY	SITRVSLKALGVALNMRVFDD-HS
CB5	ICSKGTRGVGGKY	SITRVSLKALGVALNMRVFDD-HS
Peat1	ICSKGTRGVGGKY	SITRVSLKALGVALNMRVFDD-HS
PP90	ICSKGTRGVGGKY	SITRVSLKALGVALNMRVFDD-HS
BF25/12	MCSTGSRGLGGKY	DSTRINLNALGTQLVMRTFND-HL
PP16	ICTTGNRGLNGKY	DSTRIELRSLGIKLVMRTFDD-TQ
PPWS1	ICTTGNRGLNGKY	DSTRIELRSLGIKLVMRTFDD-TQ

## Supplementary Materials

The following are available online at http://www.mdpi.com/1999-4915/10/8/394/s1, Supplementary Information 1, Table S1: Bacteria strains used in the isolation and the testing of host range of *Pectobacterium* phages CB5, Supplementary Information 1, Table S2: Details of proteins used in the phylogenetic analysis of 52 phages from the subfamily *Autographivirinae* and *Pectobacterium* phage CB5, Supplementary Information 2, Table S3: Annotation of *Pectobacterium* phage CB5, Supplementary Information 1, Table S4: Taxonomy output from VICTOR analysis of 52 phages from the subfamily *Autographivirinae* and *Pectobacterium* phage CB5, Supplementary Information 1, Table S5: Shared early region ORFs between PhiM1-like phages as determined by CoreGenes, Supplementary Information 1, Table S6: Proteins of PhiM1-like phage involved in DNA replication, repair and related metabolism, Supplementary Information 1, Table S7: Structural proteins of the PhiM1-like phages, Supplementary Information 1, Table S8: Proteins of lysis cassette of the PhiM1-like phages, Supplementary Information 1, Figure S1: *Pectobacterium* phage CB5 plaque morphology on 0.4% w/v LB overlay, Supplementary Information 1, Figure S2: Graphic showing the distribution of reads when mapped back to the contig representing the genome of *Pectobacterium* phage CB5 obtained from genome assembly, Supplementary Information 1, Figure S3: Phylogenetic analyses of amino sequences of the head–tail connecting protein, terminase, tail tube protein A, and tail tube protein B of *Pectobacterium* phage CB5 and 52 members of the *Autographivirinae* subfamily.
