# Supplementary material for: Pectobacterium atrosepticum Phage vB_PatP_CB5: A Member of the Proposed Genus ‘Phimunavirus’
Source: Viruses. 2018 Jul 26;10(8):394. doi: 10.3390/v10080394 (PMC6115819; doi:10.3390/v10080394)
Supplement: Supplementary file 1 [file viruses-10-00394-s001.zip › Buttimer et al, Viruses, supplementary information 1.docx]

Table S1. Bacteria strains used in the isolation and the testing of host range of *Pectobacterium* phages CB5

| **Bacteria** | **Strain** | **Isolation source** |
| --- | --- | --- |
| *Pectobacterium atrosepticum* | DSM 18077 (type strain) | Potato (*Solanum tuberosum*) |
|  | DSM 30184 | Potato (*Solanum tuberosum* cv. Bodenkraft) |
|  | DSM 30185 | Potato (*Solanum tuberosum*) |
|  | DSM 30186 | Potato (*Solanum tuberosum* cv. Maritta) |
|  | CB BL1-1 | *Solanum tuberosum* cv. British Queen |
|  | CB BL2-1 | Solanum tuberosum cv. British Queen |
|  | CB BL3-1 | *Solanum tuberosum* cv. British Queen |
|  | CB BL4-1 | *Solanum tuberosum* cv. British Queen |
|  | CB BL5-1 | Solanum tuberosum cv. British Queen |
|  | CB BL7-1 | Solanum tuberosum cv. Golden wonder |
|  | CB BL9-1 | Solanum tuberosum cv. Golden wonder |
|  | CB BL11-1 | *Solanum tuberosum cv.* Rooster |
|  | CB BL12-2 | *Solanum tuberosum* cv. Golden wonder |
|  | CB BL13-1 | *Solanum tuberosum* cv. Golden wonder |
|  | CB BL14-1 | *Solanum tuberosum* cv. Golden wonder |
|  | CB BL15-1 | *Solanum tuberosum* cv. Golden wonder |
|  | CB BL16-1 | *Solanum tuberosum* cv. Golden wonder |
|  | CB BL18-1 | *Solanum tuberosum* cv. Golden wonder |
|  | CB BL19-1 | *Solanum tuberosum* cv. Golden wonder |
| *Pectobacterium carotovorum subsp. carotovorum* | DSMZ 30168 (type strain) | Potato (*Solanum tuberosum*) |
|  | DSMZ 30169 | *Brassica oleracea* var.*capitata* |
|  | DSMZ 30170 | Potato (*Solanum tuberosum* "Maritta") |
|  | CB BL19-1-37 | *Solanum tuberosum* cv. Golden wonder |
| *Dickeya chrysanthemi bv chrysanthemi* | LMG 2804 (type strain) | *Chrysanthemum* |
| *Dickeya dianthicola* | PD 482 | *Solanum tuberosum* cv. Ostara |
|  | PD 2174 | - |
|  | GBBC 1538 | - |
| *Dickeya solani* | sp. PRI 2222 | - |
|  | LMG 25865 | *Solanum tuberosum* cv. Première |
|  | GBBC 1502 | - |
|  | GBBC 1586 | - |

Table S2 – Details of proteins used in the phylogenetic analysis of 52 phages from the subfamily *Autographivirinae* and *Pectobacterium* phage CB5

| **Phage** | **Genome accession no.** | **Major capsid accession no.** |
| --- | --- | --- |
| Acinetobacter phage Abp1 | NC_021316.1 | YP_008058231.1 |
| Acinetobacter phage Fri1 | NC_028848.1 | YP_009203047.1 |
| Acinetobacter phage Petty | NC_023570.1 | YP_009006529.1 |
| Acinetobacter phage phiAB1 | NC_028675.1 | YP_009189372.1 |
| Acinetobacter phage vB_ApiP_P2 | MF033351.1 | ASN73550.1 |
| Aeromonas phage phiAS7 | NC_019528.1 | YP_007007808.1 |
| Cronobacter phage vB_CskP_GAP227 | NC_020078.1 | YP_007348355.1 |
| Dickeya phage BF25/12 | KT240186.1 | ALA46504.1 |
| Enterobacteria phage J8-65 | NC_025445.1 | YP_009101383.1 |
| Enterobacteria phage K30 | NC_015719.1 | YP_004678755.1 |
| Enterobacteria phage T7 | NC_001604.1 | NP_041997.1 |
| Erwinia amylovora phage Era103 | NC_009014.1 | YP_001039668.1 |
| Escherichia phage phiKT | NC_019520.1 | YP_007006600.1 |
| Escherichia virus K1-5 | NC_008152.1 | YP_654132.1 31 |
| Escherichia virus K1E | NC_007637.1 | YP_425009.1 |
| Klebsiella phage F19 | NC_023567.2 | YP_009006057.1 |
| Klebsiella phage K11 | NC_011043.1 | YP_002003823.1 |
| Klebsiella phage KP32 | NC_013647.1 | YP_003347548.1 |
| Klebsiella phage KP34 | NC_013649.2 | YP_003347636.1 |
| Klebsiella phage NTUH-K2044-K1-1 | NC_025418.1 | YP_009098373.1 |
| Klebsiella phage vB_KpnP_SU503 | NC_028816.1 | YP_009199922.1 |
| Klebsiella phage vB_KpnP_SU552A | NC_028870.1 | YP_009204828.1 |
| Kluyvera phage Kvp1 | FJ194439.1 | ACJ14590.1 |
| Pantoea phage LIMElight | NC_019454.1 | YP_007002894.1 |
| Pantoea phage LIMEzero | NC_015585.1 | YP_004539113.1 |
| Pectobacterium phage PhiM1 | JX290549.1 | AFQ22523.1 |
| Pectobacterium phage PP16 | NC_031068.1 | YP_009286812.1 |
| Pectobacterium phage PP90 | NC_031096.1 | YP_009289647.1 |
| Pectobacterium phage PPWS1 | LC063634.2 | BAS69556.1 |
| Pectobacterium_phage_Peat1 | NC_029081.1 | YP_009224669.1+YP_009224670.1 |
| Pseudomonad phage gh-1 | AF493143.1 | AAO73167.1 |
| Pseudomonas phage Bf7 | NC_016764.1 | YP_005098192.1 |
| Pseudomonas phage LKA1 | NC_009936.1 | YP_001522884.1 |
| Pseudomonas phage LKD16 | NC_009935.1 | YP_001522824.1 |
| Pseudomonas phage LUZ19 | NC_010326.1 | YP_001671977.1 |
| Pseudomonas phage MPK6 | NC_022746.1 | YP_008766800.1 |
| Pseudomonas phage MPK7 | NC_022091.1 | YP_008431345.1 |
| Pseudomonas phage phi-2 | NC_013638.1 | YP_003345495.1 |
| Pseudomonas phage phikF77 | NC_012418.1 | YP_002727855.1 |
| Pseudomonas phage phiKMV | NC_005045 | NP_877471.1 |
| Pseudomonas phage PT2 | NC_011107.1 | YP_002117817.1 |
| Pseudomonas phage PT5 | EU056923.1 | ABW23115.1 |
| Ralstonia phage RSB1 | NC_011201.1 | YP_002213721.1 |
| Ralstonia phage RSB3 | NC_022917.1 | YP_008853924.1 |
| Ralstonia virus phiAp1 | KY117485.1 | APU03181.1 |
| Salmonella phage SP6 | NC_004831.2 | NP_853592.1 |
| Vibrio phage VP93 | NC_012662 | YP_002875653.1 |
| Xanthomonas phage f20-Xaj | KU595432.1 | AMM44667.1 |
| Xanthomonas phage f30-Xaj | KU595433.1 | AMM44714.1 |
| Xylella phage Prado | NC_022987.1 | YP_008859419.1 |
| Yersinia phage phi80-18 | HE956710.2 | CCI88880.2 |
| Yersinia phage phiR8-01 | HE956707.2 | CCI88417.2 |
| Pectobacterium phage vB_Pat_CB5 | KY953156 | ARW59018 |

Table S4. Taxonomy output from VICTOR analysis of 52 phages from the subfamily *Autographivirinae* and *Pectobacterium* phage CB5, OPTSIL clustering of taxon boundaries based on the D4 formula at genus and subfamily level. Numbers represent the genus and subfamily which phages have been allocated.

| **Phage** | **genus** | **subfamily** |
| --- | --- | --- |
| Enterobacteria phage SP6 (NC_004831) | 1 | 1 |
| Escherichia virus K1E (NC_007637) | 1 | 1 |
| Escherichia virus K1-5 (NC_008152) | 1 | 1 |
| Erwinia amylovora phage Era103 (NC_009014) | 1 | 1 |
| Pseudomonad phage gh-1 (AF493143) | 3 | 2 |
| Kluyvera phage Kvp1 (FJ194439) | 3 | 2 |
| Enterobacteria phage T7 (NC_001604) | 3 | 2 |
| Klebsiella phage K11 (NC_011043) | 3 | 2 |
| Klebsiella phage KP32 (NC_013647) | 3 | 2 |
| Escherichia phage K30 (NC_015719) | 3 | 2 |
| Ralstonia phage RSB1 (NC_011201) | 2 | 3 |
| Pseudomonas phage Bf7 (NC_016764) | 4 | 3 |
| Escherichia phage phiKT (NC_019520) | 5 | 3 |
| Yersinia phage phiR8-01 (HE956707) | 6 | 3 |
| Yersinia phage phi80-18 (HE956710) | 6 | 3 |
| Aeromonas phage phiAS7 (NC_019528) | 6 | 3 |
| Cronobacter phage vB_CskP_GAP227 (NC_020078) | 6 | 3 |
| Xanthomonas phage f20-Xaj (KU595432) | 9 | 3 |
| Xanthomonas phage f30-Xaj (KU595433) | 9 | 3 |
| Xylella phage Prado (NC_022987) | 9 | 3 |
| Pseudomonas phage PT5 (EU056923) | 7 | 4 |
| Pseudomonas phage phiKMV (NC_005045) | 7 | 4 |
| Pseudomonas phage LKD16 (NC_009935) | 7 | 4 |
| Pseudomonas phage LKA1 (NC_009936) | 7 | 4 |
| Pseudomonas phage LUZ19 (NC_010326) | 7 | 4 |
| Pseudomonas phage PT2 (NC_011107) | 7 | 4 |
| Pseudomonas phage phikF77 (NC_012418) | 7 | 4 |
| Pseudomonas phage phi-2 (NC_013638) | 7 | 4 |
| Pseudomonas phage MPK7 (NC_022091) | 7 | 4 |
| Pseudomonas phage MPK6 (NC_022746) | 7 | 4 |
| Ralstonia virus phiAp1 (KY117485) | 8 | 4 |
| Ralstonia phage RSB3 (NC_022917) | 8 | 4 |
| Pantoea phage LIMEzero (NC_015585) | 10 | 4 |
| Enterobacteria phage J8-65 (NC_025445) | 10 | 4 |
| Acinetobacter phage vB_ApiP_P2 (MF033351) | 11 | 4 |
| Acinetobacter phage Abp1 (NC_021316) | 11 | 4 |
| Acinetobacter phage Petty (NC_023570) | 11 | 4 |
| Acinetobacter phage phiAB1 (NC_028675) | 11 | 4 |
| Acinetobacter phage Fri1 (NC_028848) | 11 | 4 |
| Vibrio phage VP93 (NC_012662) | 12 | 4 |
| Klebsiella phage KP34 (NC_013649) | 12 | 4 |
| Pantoea phage LIMElight (NC_019454) | 12 | 4 |
| Klebsiella phage F19 (NC_023567) | 12 | 4 |
| Klebsiella phage NTUH-K2044-K1-1 (NC_025418) | 12 | 4 |
| Klebsiella phage vB_KpnP_SU503 (NC_028816) | 12 | 4 |
| Klebsiella phage vB_KpnP_SU552A (NC_028870) | 12 | 4 |
| Pectobacterium phage PhiM1 (JX290549) | 13 | 4 |
| Dickeya phage BF25/12 (KT240186) | 13 | 4 |
| Pectobacterium phage PPWS1 (LC063634) | 13 | 4 |
| Pectobacterium phage Peat1 (NC_029081) | 13 | 4 |
| Pectobacterium phage PP16 (NC_031068) | 13 | 4 |
| Pectobacterium phage PP90 (NC_031096) | 13 | 4 |
| Pectobacterium phage CB5 (KY953156) | 13 | 4 |

Table S5. Shared early region ORFs between PhiM1-like phages as determined by CoreGenes

| no. | Early gene region | Peat1 | CB5 | phiM1 | PP90 |
| --- | --- | --- | --- | --- | --- |
| 1 | hypothetical protein | AXI77_gp61 | CB5_3 | PhiM1_03 | PP90_2 |
| 2 | hypothetical protein | AXI77_gp01 | CB5_6 | PhiM1_04 | PP90_3 |
| 3 | hypothetical protein | AXI77_gp07 | CB5_12 | PhiM1_08 | PP90_7 |
| 4 | hypothetical protein | AXI77_gp08 | CB5_13 | PhiM1_09 | PP90_8 |
| 5 | peptidase | AXI77_gp09 | CB5_15 | PhiM1_10 | PP90_9 |
| 6 | hypothetical protein | AXI77_gp10 | CB5_16 | PhiM1_11 | PP90_10 |

Table S6. Proteins of PhiM1-like phage involved in DNA replication, repair and related metabolism

| **no.** | **DNA replication & nucleotide metabolism** | **Peat1** | **CB5** | **phiM1** | **PP90** |
| --- | --- | --- | --- | --- | --- |
| 1 | DNA primase | AXI77_gp12 + AXI77_gp13 | CB5_18 | PhiM1_12 | PP90_12 |
| 2 | DNA helicase | AXI77_gp17 | CB5_22 | PhiM1_14 | PP90_15 |
| 3 | DNA polymerase | AXI77_gp21 | CB5_28 | PhiM1_14 | PP90_21 |
| 4 | 5' exonuclease | AXI77_gp23 | CB5_31 | PhiM1_22 | PP90_24 |
| 5 | endonuclease VII | AXI77_gp25 | CB5_34 | PhiM1_25 | PP90_27 |
| 6 | DNA dependent RNA polymerase | AXI77_gp30 | CB5_39 | PhiM1_31 | PP90_32 |
| 7 | 5' kinase / 3'phosphatase | _ | _ | PhiM1_28 | PP90_29 |
| 8 | nucleatidyl transferase | _ | CB5_24 | _ | _ |

Table S7. Structural proteins of the PhiM1-like phages

| **no.** | **Structural protein** | **Peat1** | **CB5** | **phiM1** | **PP90** |
| --- | --- | --- | --- | --- | --- |
| 1 | head tail connector | AXI77_gp34 | CB5_44 | PhiM1_35 | PP90_38 |
| 2 | scaffolding protein | AXI77_gp37 | CB5_45 | PhiM1_36 | PP90_40 +PP90_41 |
| 3 | major capsid | AXI77_gp38+AXI77_gp39 | CB5_46 | PhiM1_38 | PP90_43 |
| 4 | tubular protein A | AXI77_gp41+AXI77_gp42 | CB5_47 | PhiM1_39 | PP90_44 |
| 5 | tubular protein B | AXI77_gp43 | CB5_49 | PhiM1_40 | PP90_45 |
| 6 | internal virion protein A | AXI77_gp44 | CB5_50 | PhiM1_41 | PP90_46 |
| 7 | internal virion protein B | AXI77_gp45 | CB5_51 | PhiM1_42 | PP90_47 |
| 8 | internal virion protein C | AXI77_gp46+AXI77_gp47 | CB5_52 | PhiM1_43 | PP90_48 |
| 9 | tail fibre | AXI77_gp48 | CB5_53 | PhiM1_44 | PP90_49 |
| 10 | large terminase | AXI77_gp50 | CB5_54 | PhiM1_45 | PP90_50 |
| 11 | small terminase | AXI77_gp51 | CB5_55 | PhiM1_46 | PP90_51 |
| 12 | tail spike | AXI77_gp57 | CB5_60 | PhiM1_52 | PP90_56 |

Table S8. Proteins of lysis cassette of the PhiM1-like phages

| **no.** | **Lysis protein** | **Peat1** | **CB5** | **phiM1** | **PP90** |
| --- | --- | --- | --- | --- | --- |
| 1 | U-spanin | AXI77_gp54 | CB5_57 | PhiM1_49 | PP90_53 |
| 2 | Holin | AXI77_gp55 | CB5_58 | PhiM1_50 | PP90_54 |
| 3 | Endolsyin | AXI77_gp56 | CB5_59 | PhiM1_51 | PP90_55 |


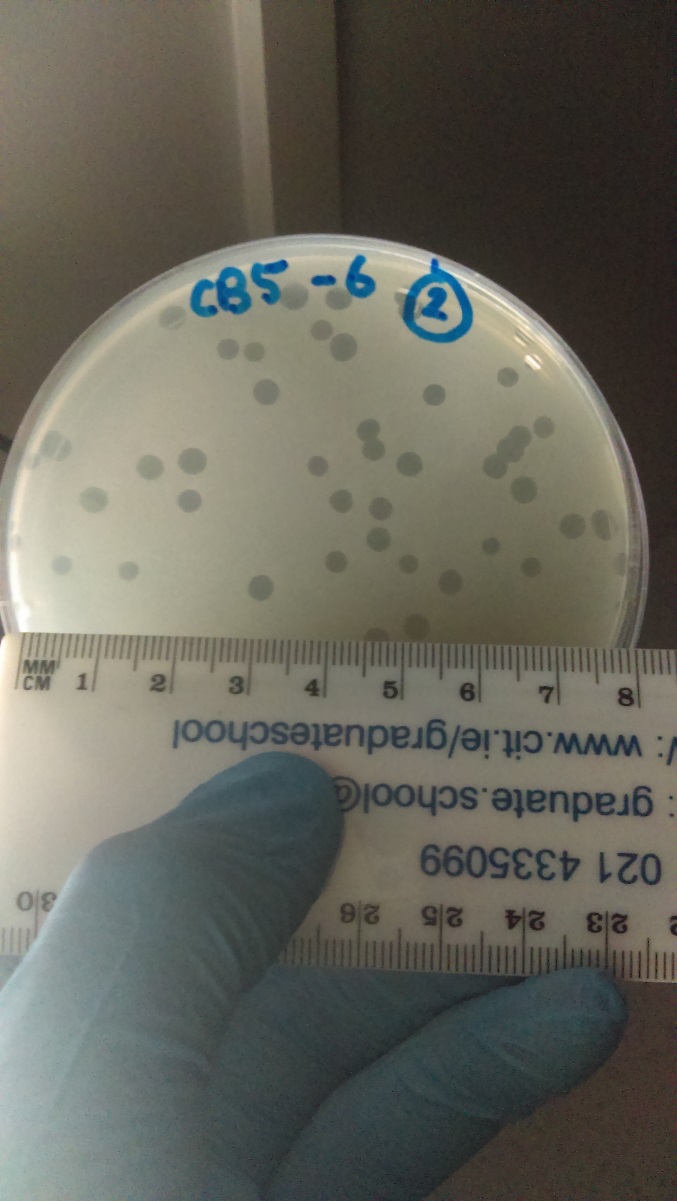


Figure S1. *Pectobacterium* phage CB5 plaque morphology on 0.4% w/v LB overlay using host strain *P. atrosepticum* DSM 30186 (12 hr incubation).


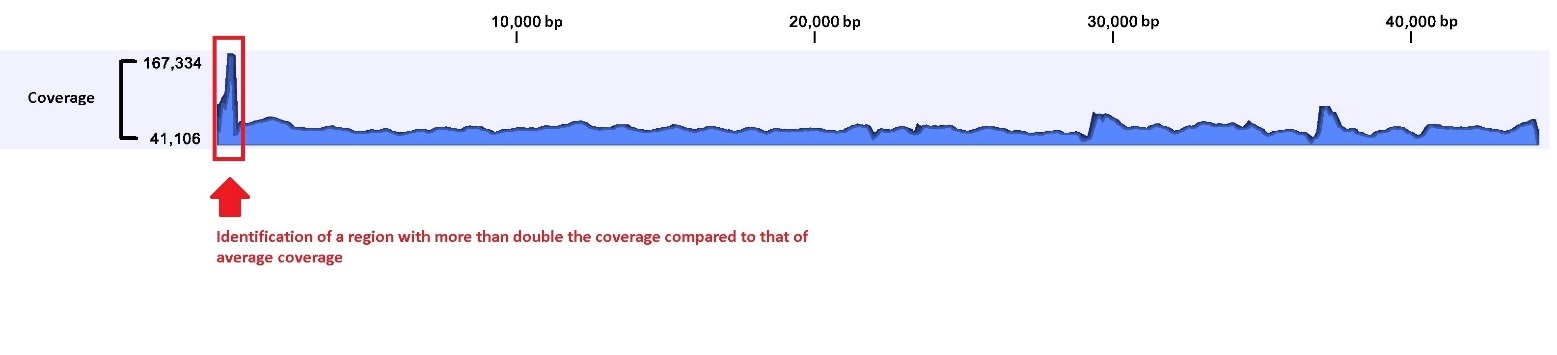


Figure S2. Coverage map showing the distribution of reads when mapped back to the contig representing the genome of *Pectobacterium* phage CB5 obtained from genome assembly. Map created with CLC Genomics Workbench.


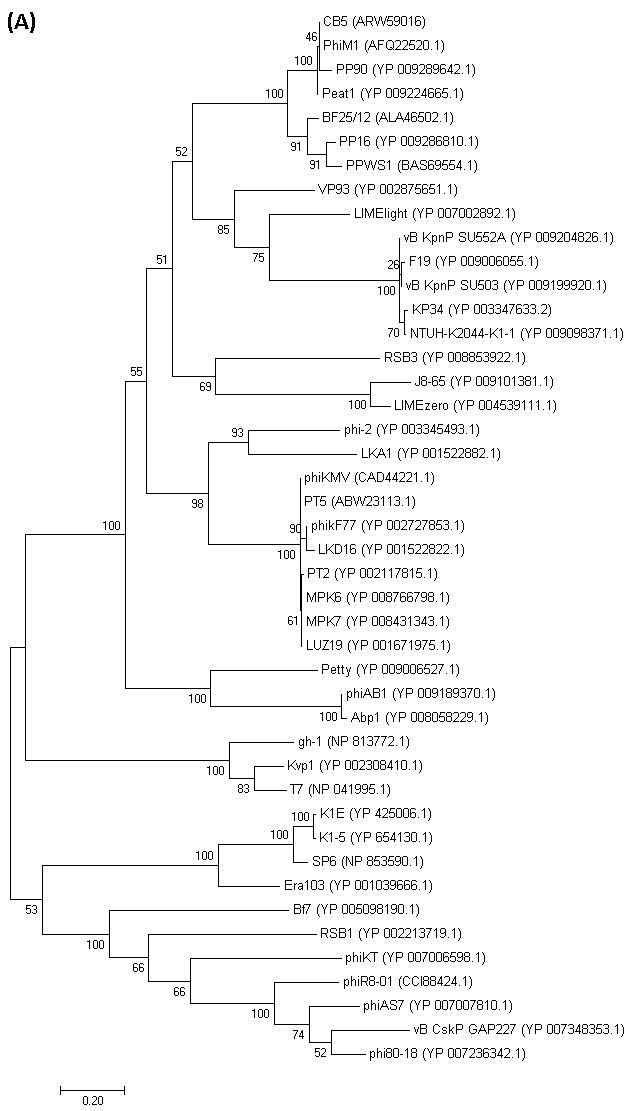


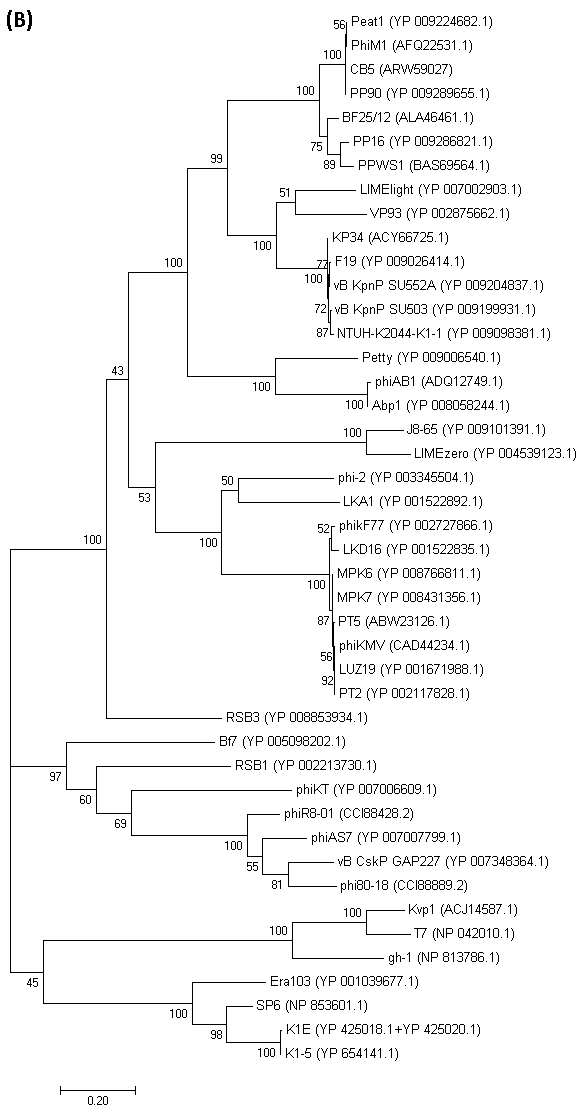


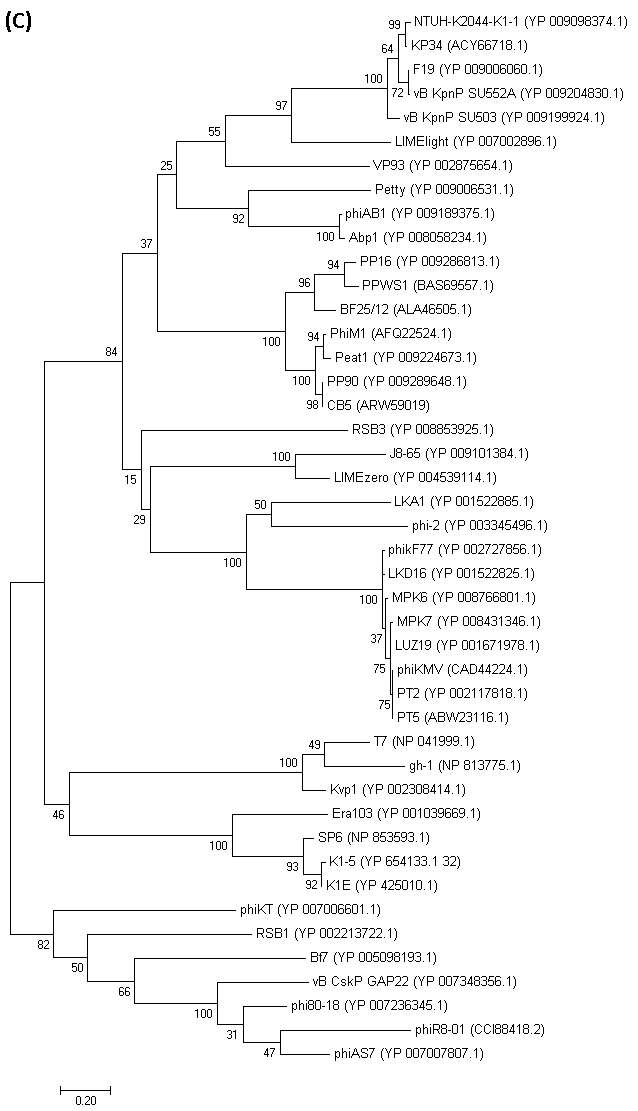


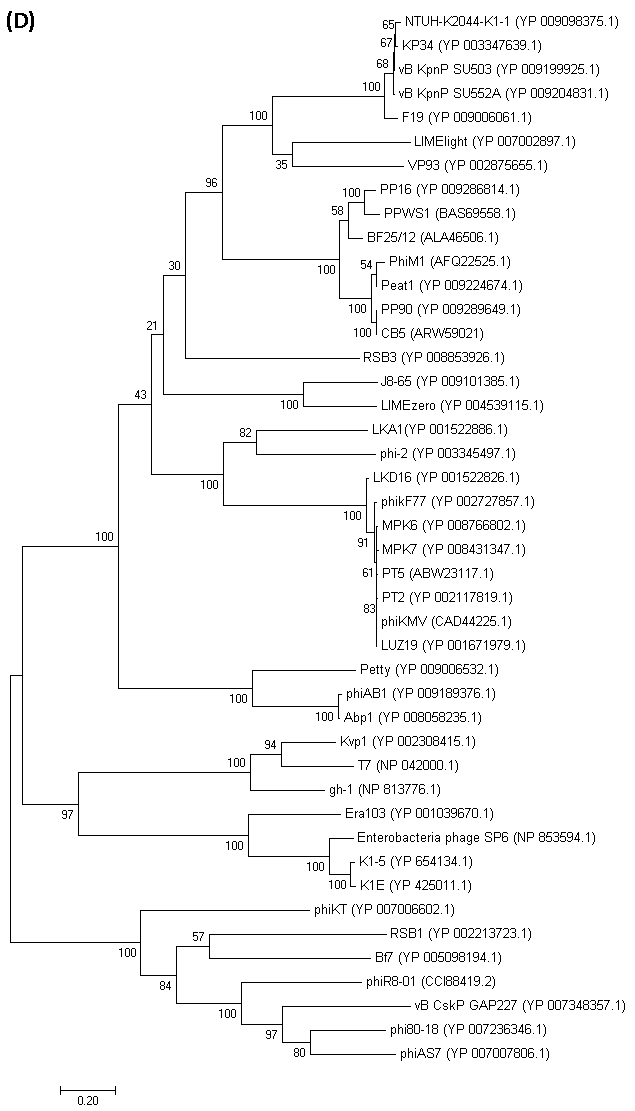


Figure S3. Phylogenetic analyses of amino sequences of the head-tail connecting protein (A), terminase (B), tail tube protein A (C) and tail tube protein B (D) of *Pectobacterium* phage CB5 and 52 members of the *Autographivirinae* subfamily, using maximum likelihood (Whelan and Goldman substitution model), with 100 bootstrap replicates.
